# Urinary Catheterization May Not Adversely Impact Quality of Life in Multiple Sclerosis Patients

**DOI:** 10.1155/2014/167030

**Published:** 2014-02-20

**Authors:** Rebecca James, Heidi E. Frasure, Sangeeta T. Mahajan

**Affiliations:** ^1^Department of Urology, Division of Female Pelvic Medicine and Reconstructive Surgery, University Hospitals Case Medical Center, 11100 Euclid Avenue, LKSD 5046, Cleveland, OH 44106, USA; ^2^University Hospitals Case Medical Center, 11100 Euclid Avenue, MAC 5034, Cleveland, OH 44106, USA; ^3^Department of OB/GYN, Division of Female Pelvic Medicine and Reconstructive Surgery, University Hospitals Case Medical Center, 11100 Euclid Avenue, MAC 5034, Cleveland, OH 44106, USA

## Abstract

*Background.* Multiple sclerosis (MS) healthcare providers (HCP) have undergone considerable educational efforts regarding the importance of evaluating and treating pelvic floor disorders, specifically, urinary dysfunction. However, limited data are available to determine the impact of catheterization on patient quality of life (QoL). *Objectives.* To describe the use of urinary catheterization among MS patients and determine the differences between those who report positive versus negative impact of this treatment on QoL. *Methods.* Patients were queried as part of the 2010 North American Research Committee On Multiple Sclerosis survey; topics included 1) urinary/bladder, bowel, or sexual problems; 2) current urine leakage; 3) current catheter use; 4) catheterizing and QoL. *Results.* Respondents with current urine leakage were 5143 (54.7%), of which 1201 reported current catheter use (12.8%). The types of catheters (intermittent self-catheterization and Foley catheter (indwelling and suprapubic)) did not differ significantly. Of the current catheter users, 304 (25.35%) respondents reported catheterization negatively impacting QoL, 629 (52.4%) reported a positive impact on QoL, and 223 (18.6%) reported neutral QoL. *Conclusions. *A large proportion of catheterized MS patients report negative or positive changes in QoL associated with urinary catheterization. Urinary catheterization does not appear to have a universally negative impact on patient QoL.

## 1. Introduction

Urinary catheterization, either indwelling or intermittent, is often utilized for the treatment of chronic voiding dysfunction associated with multiple sclerosis (MS). To the outside observer, urinary catheterization may often be feared as “painful” or “uncomfortable” and presumed to negatively impact patient quality of life (QoL). Unfortunately, for patients with MS and other neurologic conditions refusing to catheterize is not an option. To date, the number of MS patients requiring this invasive therapy has remained unknown [1].

Our unit previously demonstrated significant rates of satisfaction with the evaluation and treatment of bladder and bowel dysfunction in patients with MS. Recent efforts to increase patient, family, and clinician awareness regarding the importance of pelvic floor issues appear to be positively received by most MS patients and to favorably impact their QoL [2]. Urinary catheterization is often an important part of the treatments for voiding disorders.

The goal of this study was to determine the prevalence of urinary catheterization among men and women with MS using a large community-dwelling population sample. Form of urinary catheterization used as well as patient characteristics were also assessed. The impact of this urinary catheterization on MS patient QoL, including positive versus negative responders, was also assessed.

## 2. Methods

The Consortium of Multiple Sclerosis Centers (http://www.mscare.org/) administer the largest registry in North America of MS patients who agree to participate in research related activities, called the North American Research Committee of Multiple Sclerosis (NARCOMS) Registry. The NARCOMS Registry captures self-reported demographic and clinical information from patients with MS at enrollment and semiannually thereafter, via questionnaires administered either online or by mail, per participant preference. The registry is the largest self-report database of patients with MS worldwide, with more than 16,000 active participants, and is approved by the Western Institutional Review Board (IRB) and the IRB at the University of Alabama at Birmingham. We obtained an exemption from University Hospitals Case Medical Center IRB for this study. Deidentified data from the Fall 2010 questionnaire was supplied for analysis by NARCOMS in the form of an excel spreadsheet with a data dictionary.

At enrollment in NARCOMS, participants provide demographic information and clinical information including age at MS onset. Each semiannual NARCOMS questionnaire includes an assessment of disability status and quality of life (QoL). The Patient Determined Disease Steps (PDDS) measures disability based on self-report and has been validated in large MS populations against the physician-scored Expanded Disability Status Scale [3–6]. It is scored from 0 to 8, with 0 defined as no disability and 8 as bed bound (barriers). Respondents are also evaluated for bladder and spasticity disability using standardized questionnaires.

In the Fall of 2010 the authors collaborated with the staff of the NARCOMS database to create a short 2-page questionnaire for inclusion in the Fall 2010 questionnaire regarding pelvic floor disorders. Specifically, patients were asked their degree of bother with urinary/bladder, bowel, and sexual problems based on a 4-point scale (1 not at all bothered, 4 severely bothered). Patients were queried as to whether their health care provider (HCP) had inquired about each type of pelvic floor complaint in the last 12 months (yes versus no). Patients were further asked to rate on a 5-point Likert scale (1 not at all, 5 completely) their satisfaction with the care they received for each complaint. Finally, they were asked if their quality of life had changed with treatment (7-point Likert scale, 1 much better, 7 much worse).

Utilization of any form of urinary catheterization was assessed, including type of catheter used (intermittent self-catheterization (ISC), suprapubic catheterization (SC), and transurethral Foley catheterization (TFC)). Medication usage, evaluation by a urologist, and correlation with disability as measured by PDDS score were also evaluated.

Data analysis used SAS version 9.1 (SAS Institute Inc., Cary, N.C.) and descriptive statistics, chi-square tests for frequency data and calculation of correlation coefficients and 95% confidence intervals were constructed as appropriate.

## 3. Results

In the Fall of 2010, the biannual NARCOMS questionnaire was delivered to 14268 participants of which 9397 (66%) responses were returned. Respondents were primarily white (89%), women (77.4%), with an average age of 55 (SD 10.5) years (Table 1). Significant disability, defined as a PDDS score ≥3 (representing gait disability or worse) was reported by 6070 (64.6%; 95% CI [63.6, 65.5]) respondents. Moderate to severe bladder disability scores were reported by 2743 (29.2%; 95% CI [28.3, 30.1]) of respondents, with 281 (3%; 95% CI [2.7, 3.4]) reporting total loss of bowel and bladder control.

Overall, 12.8% of all respondents reported current urinary catheterization use (Table 2). The types of catheters used by respondents included intermittent self-catheterization, Foley catheter (indwelling), and suprapubic. Intermittent catheterization was by far the most commonly used method reported by patients (*P* < 0.005). Patients who reported urinary incontinence were more likely to utilize urinary catheterization. Greater than fifty percent of all respondents (5143 (54.7%)) reported some current urinary leakage complaints. Among these incontinent patients, 1201 (23.4%) reported current catheter use.

Urinary catheter use had an unpredictable impact on QoL, although most patients reported the impact to be positive. Among the current catheter users (*n* = 1193), 629 (52.4%) respondents reported catheterization having a positive impact on QoL, 304 (25.3%) reported a negative impact, and 260 (21.8%) reported neutral impact (Table 3). Type of catheter used did not appear to predict impact QoL, as equal numbers of patients reporting positive, negative or neutral impact of catheterization on QOL used each form of catheter (Table 3, *P* = 0.631).

The majority of patients reported a positive impact of catheter use on their QoL (Table 4). Using a 7-point Likert scale, patients graded the impact of urinary catheterization on their QoL. The largest proportion of patients reported a “very positively/positively/slightly positively” impact on QoL.

Increasing degree of physical disability did not predict positive, negative, or neutral impact of catheter use on QoL. Using a PDDS score of >3 as a marker of significant disability, equal numbers of patients reported positive, negative or neutral impact of catheter use on their QoL (Table 3). However, as patient disability continued to progress (PDDS score 3–5 versus 6–8), greater numbers of patients reported using urinary catheterization as a treatment method and tended to be satisfied with their care for this complaint (*P* = 0.004 and *P* < 0.001,Table 5). Age did not appear to impact satisfaction rates with respondents ≤55 years compared to >55 years of age who reported a similar level of satisfaction (very/completely) for bladder complaints (*P* = 0.202).

## 4. Discussion

Among the MS patient population, urinary catheterization is common. Given that 25–56% of MS patients suffer from obstructive phase voiding dysfunction, catheterization remains a useful and necessary treatment choice for patients and their providers [7].

While some studies have evaluated QoL in and barriers to catheter use in nonspecific patient populations, little attention has been awarded to the patient-oriented impact of catheter use in the MS population [8]. Our large database of MS patients with a questionnaire response rate of 66% allows specific investigation into the question of catheterization and QoL in MS. Our results demonstrate that catheterization may not adversely impact QoL in MS patients. Large proportions of catheterized MS patients report either negative or positive changes in QoL associated with urinary catheterization, suggesting that there is no clear positive or negative impact of this treatment. Patients with higher bladder distress symptom and spasticity scores tended to report a negative impact on QoL, although this difference could be related to their more severe MS disease state, higher MS relapse rate, or both. Similar findings were noted by Bolinger et al. reporting that patients with reduced manual dexterity had increased difficulty with intermittent catheter insertion and dissatisfaction with this therapy [8]. In general, more severely affected patients may tend to report increasingly negative impact on QoL.

Patient PDDS scores demonstrated a trend towards significance with regards to positive versus negative impact of catheterization on QoL. Again, the more severely affected patients tended to report a higher PDDS score (more severe disease state). Consistently, as PDDS scores increased, more patients appeared to view catheterization as a helpful treatment (positive QoL impact). Type of catheter used did not significantly impact QoL in these respondents.

Urinary catheterization does not appear to have the universally negative impact on patient QoL that is often assumed to accompany this treatment. Given the importance of urinary catheterization to the management of daily lives of a significant proportion of MS patients, closer examination of the predictors of positive or negative QoL is advantageous. An improved understanding of how to optimize a catheterizing patient's QoL can assist healthcare providers in delivering a higher quality of care. More specific predictors as well as patient opinions associated with catheter use in MS patients should be explored to further delineate contributions to negative or positive QoL.

## Figures and Tables

**Table 1:** Patient demographics.

Gender	
Female	7271 (77.4%)
Male	2126 (22.6%)
Race	
Caucasian	8363 (89%)
African-american	203 (2.2%)
Hispanic/latino	136 (1.4%)
Other	241 (2.6%)
Unknown (not answered)	454 (4.8%)
Age	
Mean (SD)	55.4 (10.5)
Range	20–93
Time since diagnosis, mean (SD)	16.5 (9.7)
Education level	
Less than 12 years	109 (1.2%)
High school	2547 (27.1%)
Technical/associate degree	2141 (22.8%)
Bachelor's degree	2434 (25.9%)
Graduate degree	2166 (23.0%)
Currently employed	2181 (23.2%)
Annual income	
1 (<15,000)	800 (8.5%)
2 (15–30 K)	1337 (14.2%)
3 ( 30–50 K)	1551 (16.5%)
4 (50–100 K)	2261 (24.1%)
5 (>100 K)	1327 (14.1%)
6 (no answer)	2121 (22.6%)
Health insurance (yes/no)	8925 (95%)

**Table 2:** Total number of patients utilizing urinary catheterization and types of catheters used.

Total number of patients reporting urinary catheter use	**1193 (12.8%)**
Catheter type:	
Intermittent	727 (64.7%)
Indwelling	169 (14.2%)
Suprapubic	101 (8.5%)
Unsure	128 (10.7%)

**Table 3:** Catheter used based on reported impact on quality of life and impact on disability, bladder complaints, and spasticity.

Group (*N*)	Reported positive change in QoL (*n* = 629)	Reported no change on QoL (*n* = 260)	Reported negative change in QoL (*n* = 304)	*P* value*
Catheterizing patients (1193)				
Intermittent (727)	387 (61.5%)	148 (56.9%)	192 (63.2%)	0.631
Indwelling (169)	87 (13.8%)	42 (16.1%)	40 (13.2%)	0.779
Suprapubic (169)	101 (16.1%)	34 (13.1%)	34 (11.2%)	0.047
Unsure (128)	54 (8.6%)	36 (13.8%)	38 (12.5%)	0.060
PDDS score ≥ 3	562 (89.3%)	218 (83.8%)	256 (84.2%)	0.025
Bladder score ≥ 3	400 (63.6%)	141 (54.2%)	225 (74.0%)	0.002
Spasticity score ≥ 3	298 (47.4%)	110 (42.3%)	176 (57.9%)	0.003

*P value for comparison between positive and negative columns.

**Table 4:** Catheterization and quality of life: the impact of catheter use on quality of life in patients who answered “yes” to current catheter use (based on 7-point Likert scale, *n* = 1193).

Patient rating	Overall
Very negatively/negatively/slightly negatively	304 (25.5%)
(Likert score = 1 to 3)	
Neutral (Likert score = 4)	260 (21.8%)
Very positively/positively/slightly positively	629 (52.7%)
(Likert score = 5 to 7)	

**Table 5:** Catheterization and disability: rates of catheter used based on disability as gauged by the Patient-Determined Disease Steps.

PDDS	Current catheterization		*P* value*
	Yes	No	
PDDS 0–2	71 (2.1%)	3244 (97.5%)	0.075
Very/completely satisfied	37/71 (52.1%)	1349/3244 (41.6%)	
PDDS 3–5	278 (8.3%)	3091 (91.7%)	0.004
Very/completely satisfied	149/278 (53.6%)	1380/3091 (44.6%)	
PDDS 6–8	835 (33.2%)	1683 (66.8%)	<0.001
Very/completely satisfied	481/835 (57.6%)	793/1683 (47.1%)	

Comparison of number of responses very/completely satisfied between current catheterization users and noncatheterizing patients for disability categories.

PDDS: Patient-Determined Disease Steps.
